# Clinical characteristics and risk factors of severe bocavirus‐positive pneumonia in children and a literature review

**DOI:** 10.1002/pdi3.2523

**Published:** 2025-02-06

**Authors:** Jing Liao, Zhongying Yang, Yu He, Jianhua Wei, Luo Ren, Enmei Liu, Na Zang

**Affiliations:** ^1^ Department of Respiratory Medicine Children's Hospital of Chongqing Medical University National Clinical Research Center for Child Health and Disorders Ministry of Education Key Laboratory of Child Development and Disorders Chongqing Key Laboratory of Child Rare Diseases in Infection and Immunity Chongqing China; ^2^ Pediatric Research Institute of Children's Hospital of Chongqing Medical University Chongqing China; ^3^ Key Laboratory of Children's Important Organ Development and Diseases of Chongqing Municipal Health Commission Chongqing China

**Keywords:** bocavirus, children, clinical features, risk factors, severe pneumonia

## Abstract

Increasing evidence suggests that human bocavirus (HBoV) is associated with respiratory symptoms in the absence of other identifiable pathogens and may even precipitate severe lower respiratory tract infections. However, only a few studies of severe human bocavirus infections in pediatric patients have been reported. The aim of the current study was to collect and analyze clinical data from children diagnosed with mild pneumonia and severe pneumonia, with an emphasis on those testing positive for HBoV from June 2009 to June 2019. Among the 799 HBoV‐positive children included in the study, approximately 5.88% experienced severe pneumonia. Results revealed no significant differences between co‐detection and single detection of HBoV‐positive pneumonia in the severe and mild groups, supporting the pathogenicity of HBoV. A higher incidence of severe cases was observed in children lacking *Bacillus* Calmette–Guérin (BCG) vaccination, or with congenital airway or bronchopulmonary dysplasia (*P* = 0.046, *P* = 0.017). Overall, these findings indicate that HBoV can be identified in respiratory samples from children with severe pneumonia, denoting its role as a viral pathogen in hospitalized children with this condition. Preterm birth, wheezing history of previous infection, pulmonary underlying diseases such as airway dysplasia or bronchopulmonary dysplasia, and viremia may be the risk factors for severe pneumonia with HBoV positive.

## Graphical abstract

The study explored the clinical characteristics and risk factors of HBoV‐positive severe pneumonia in children. These findings indicate that HBoV can be identified in respiratory samples from children with severe pneumonia, denoting its role as a viral pathogen in hospitalized children with this condition.

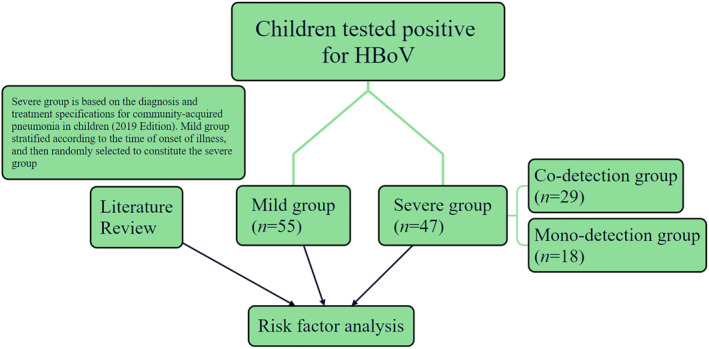

## 1 INTRODUCTION

Human bocavirus (HBoV) was initially isolated from respiratory specimens in 2005,^1^ a new member of the *Parvoviridae*. Subsequent research recognized three additional genotypes of HBoV (HBoV2, 3, and 4) in fecal samples.^2^ HBoV1, a single‐stranded DNA parvovirus, is implicated in respiratory diseases, including lower respiratory tract infections and acute wheezing in infants and young children, as well as in gastrointestinal infections.^3^ Current research suggests that HBoV triggers inflammation by inducing pyroptosis in host airway epithelial cells, resulting in lung tissue injury in patients with HBoV infection.^4^ Various HBoV proteins, such as nuclear phosphoprotein 1 (NP1), nonstructural protein 1 (NS1), and viral capsid protein 2 (VP2), are known to suppress the host immune system.^5^ HBoV primarily induces immune escape by inhibiting the type I interferon (IFN) signaling pathway in host cells.^5^ Zhang et al.^6^ reported frequent detection of HBoV in children with acute lower respiratory tract infections in China and noted the common occurrence of co‐infection, especially in infants and young children in intensive care units (ICU). Nascimento‐Carvalho et al.^7^ reported that among 159 polymerase chain reaction (PCR)‐positive cases of non‐severe community‐acquired pneumonia in Brazil, 38 were serologically confirmed to have acute HBoV1 infection. Choi et al.^8^ identified 11 cases of HBoV‐related severe pneumonia in adults in South Korea, representing 0.5% of severe pneumonia cases, with predominance in patients with immunodeficiencies or structural lung diseases. Furthermore, Ji et al.^9^ observed a higher incidence of severe pneumonia in HBoV‐positive patients than in HBoV‐negative patients in Ningxia, China, indicating a potential link between HBoV infection and the severity of pneumonia in the positive group. In recent years, various countries have reported cases of severe acute lower respiratory tract infection and even death resulting from single HBoV infection.^10^, ^11^ Ogimi et al.^12^ identified young age and a history of HBoV exposure as risk factors for infection in 51 pediatric and 420 adult patients undergoing hematopoietic stem cell transplantation. Despite these findings, reports on the clinical characteristics and risk factors of HBoV‐positive severe pneumonia in children remain limited. Thus, this study described the clinical features of HBoV‐positive severe pneumonia in pediatric patients in Chongqing of China through a retrospective study, and analyzed the associated risk factors, to understand HBoV as a causative pathogen.

## 2 MATERIALS AND METHODS

### 2.1 Participants

Children hospitalized for acute lower respiratory tract infection at the Children's Hospital of Chongqing Medical University (Chongqing, China) from June 2009 to June 2019, and who had HBoV‐positive respiratory tract specimens, were included in this study. Diagnosis of severe pneumonia was based on the diagnosis and treatment specifications for community‐acquired pneumonia in children (2019 Edition).^13^ The criteria of severe pneumonia definition were as follows: (1) the general situation is poor; (2) disturbance of consciousness; (3) hypoxemia: cyanosis, rapid breathing (Infants breathe at a rate of no less than 70 times per minute. Children over 1 year old breathe at a rate of no less than 50 times per minute), assisted breathing (such as groaning, nasal flaring, and three concave signs), intermittent apnea, and oxygen saturation less than 92%; (4) fever: high fever or persistent fever lasting more than 5 days; (5) dehydration or refusal to eat; (6) chest X‐ray or chest CT scan shows more than 2/3 of one lung infiltrated, multilobar lung infiltration, pleural effusion, pneumothorax, atelectasis, lung necrosis, and lung abscess; and (7) development of extrapulmonary complications. The hospitalized children in the mild disease group were stratified according to the time of onset of illness, and then randomly selected to constitute the control group. Exclusion criteria included existing active infection in other organs, preexisting organ dysfunction, malignant tumor, and incomplete clinical data. This study was approved by the Medical Ethics Committee of the Children's Hospital of Chongqing Medical University (Ethics Approval [Research] No. [217‐1], 2019).

### 2.2 Measures

At admission, specialist nurses collected nasopharyngeal secretions from the patients, which were initially stored at 4°C in a refrigerator and subsequently stored at −80°C after packaging and processing, pending further analysis. Multiplex PCR was used to detect common respiratory viruses in the nasopharyngeal aspirates (NPAs) using a QIAamp MinElute Virus Spin Kit (Qiagen, Hilden, Rhineland‐Palatinate, Germany). The samples were analyzed using commercial detection kits from TaKaRa Biotechnology (RR01AM, Dalian, Liaoning Province, China) and Applied Biosystems (Carlsbad, California, USA), targeting respiratory syncytial virus (RSV), influenza virus (IFV), parainfluenza virus (PIV), HBoV, human adenovirus (HAdV), human rhinovirus (HRV), human coronavirus (HCoV), and human metapneumovirus (HMPV). Nasopharyngeal aspirate was collected for bacterial culture and *Mycoplasma pneumoniae* PCR testing, while blood samples were taken for *Mycoplasma pneumoniae* IgM antibody testing. All procedures were conducted in accordance with the provided instructions.

### 2.3 Statistical analysis

Measurement data with normal distribution were expressed as mean ± standard deviation (x̄ ± s). An independent sample *t*‐test was used to compare measurement data with approximately normal distribution and homogeneity of variance between groups; otherwise, nonparametric tests were used. Enumeration data were expressed as percentages, and comparisons between groups were performed using the chi‐squared test or Fisher’s exact test. Univariate and multivariate logistic regression analyses were performed for each index. SPSS v20.0 software was used for statistical analysis, and *P* < 0.05 was considered statistically significant.

## 3 RESULTS

### 3.1 Demographic characteristics and epidemic season of HBoV‐positive severe pneumonia in children

From June 2009 to June 2019, a total of 799 children tested positive for HBoV DNA in their NPA samples, including 47 severe cases (5.88%). In the severe group, five patients (5/47, 10.6%) were transferred to the ICU, four patients (4/47, 8.5%) underwent mechanical ventilation, and one patient (1/47, 2.1%) died. Patients with HBoV‐positive severe pneumonia were predominantly aged between 6 months and 2 years (*n* = 32), accounting for 68.1% of total HBoV‐positive severe pneumonia cases, followed by children ≤6 months (*n* = 10), accounting for 21.2% of total cases. Only five cases were older than 2 years, accounting for 10.6%. There were 31 males and 16 females, with a male‐to‐female ratio of 1.94:1, with an average age of 10 months. Of the patients with HBoV‐positive mild pneumonia, 10 cases (10/55,18.2%) were ≤6 months, 7 cases (7/55,12.7%) were over 2 years, and the rest were between 6 months and 2 years. The positive rate of HBoV was higher in summer, with epidemic peaks in 2011, 2012, and 2013, accounting for 45.5%, 44.4%, and 57.1%, respectively, of total HBoV‐positive pneumonia cases in that year.

### 3.2 Clinical features in HBoV‐positive children with severe or mild pneumonia

In total, 47 children with HBoV‐positive severe pneumonia were enrolled in the case group, and 55 children with HBoV‐positive mild pneumonia were enrolled in the control group. There were no significant differences in sex, age, low birth weight, premature birth, and history of birth asphyxia between the severe and mild groups (*P* > 0.05). However, there was a significant difference in *Bacillus* Calmette–Guérin (BCG) vaccination, with seven (7/47, 14.9%) children in the severe group and two (2/55, 3.6%) children in the mild group not receiving the vaccination (*P* = 0.046). The course of the disease did not differ significantly between the two groups, although the severe group had a significantly longer stay at the hospital than the mild group (*P* = 0.000). The severe group also had a higher proportion of patients with fundamental diseases, particularly airway or bronchopulmonary dysplasia (21.3% *vs*. 5.5%, *P* = 0.017). There were no significant differences in history of infection, congenital heart disease, and malnutrition between the two groups (*P* > 0.05). For additional details, Table 1 provides a comparison of general data between the two groups.

**TABLE 1:** Comparison of demographic data between HBoV‐positive mild and severe pneumonia cases (*n* [%]).

	Severe group (*n* = 47)	Mild group (*n* = 55)	χ^2^/*z*/*t*	*P*
Sex				
Male	31 (66.0%)	41 (74.5%)	0.900	0.343
Female	16 (34.0%)	14 (25.5%)		
Age [M(Q1, Q3), m]	10 (7, 15)	9 (6, 20)	−0.316	0.752
Low birth weight	10 (21.3%)	6 (10.9%)	2.060	0.151
Premature birth	8 (17.0%)	6 (10.9%)	0.800	0.371
Asphyxia	4 (8.5%)	7 (12.7%)	0.468	0.494
Without BCG	7 (14.9%)	2 (3.6%)	3.992	0.046
Recurrent infection	32 (68.1%)	32 (58.2%)	1.063	0.302
Fundamental disease				
Airway or bronchopulmonary dysplasia	10 (21.3%)	3 (5.5%)	5.705	0.017
Congenital heart disease	10 (21.3%)	5 (9.1%)	3.000	0.083
Malnutrition	3 (6.4%)	0 (0%)	3.617	0.057
Course of disease [M(Q1, Q3), d]	6 (3, 14)	10 (5, 15)	−1.658	0.097
Length of stay [M(Q1, Q3), d]	9 (7, 11)	6 (5, 7)	−5.294	0.000

In this study, among the 102 children who tested positive for HBoV, 57 (55.9%) exhibited wheezing, 37 (36.3%) had diarrhea, and 101 (99.0%) presented with a cough. No significant differences in the frequency of cough, wheezing, or gastrointestinal symptoms were observed between the severe and mild groups (*P* > 0.05). However, the severe group had a higher likelihood of experiencing fever (27 cases, 57.4%), shortness of breath (16 cases, 34.0%), nasal flaring (10 cases, 21.3%), inspiratory retractions (28 cases, 59.6%), lung rales (46 cases, 97.9%) and wheezing rale (32 cases, 68.1%) than the mild group. The incidences of atelectasis or bronchiectasis (*P* = 0.039) and blood (*P* = 0.026), cardiovascular (*P* = 0.026), and nervous system disorders (*P* = 0.046) were significantly higher in the severe group than the mild group. Table 2 displays the clinical manifestations and complications for both groups.

**TABLE 2:** Comparison of clinical manifestations and complications of HBoV‐positive mild and severe pneumonia (*n* [%]).

	Severe group (*n* = 47)	Mild group (*n* = 55)	χ^2^/*z*/*t*	*P*
Manifestation				
Cough	47 (100.0%)	54 (98.2%)	0.863	0.353
Wheezing	27 (57.4%)	30 (54.5%)	0.087	0.769
Tachypnea	16 (34.0%)	5 (9.1%)	9.651	0.002
Fever	27 (57.4%)	19 (34.5%)	5.368	0.021
Diarrhea	17 (36.2%)	20 (36.4%)	0.000	0.984
Signs				
Moist rales	46 (97.9%)	47 (85.5%)	4.858	0.028
Wheezing rale	32 (68.1%)	26 (47.3%)	4.475	0.034
Nasal flaring/Inspiratory retraction	28 (59.6%)	1 (1.8%)	41.547	0.000
Cyanosis	34 (72.3%)	31 (56.4%)	2.798	0.094
Complications				
Atelectasis or bronchiectasis	7 (14.9%)	0 (0%)	4.012	0.039
Cardiovascular	12 (25.5%)	5 (9.1%)	4.932	0.026
Digestive	19 (40.4%)	20 (36.4%)	0.177	0.674
Nervous	7 (14.9%)	2 (3.6%)	3.992	0.046
Hematologic	12 (25.5%)	5 (9.1%)	4.932	0.026
Electrolyte disturbance	7 (14.9%)	4 (7.3%)	1.530	0.216

Regarding laboratory tests, hemoglobin (Hb) (109.02 ± 15.55 *vs.* 115.38 ± 13.21, *P* = 0.028) and albumin (ALB) (42.5 (38.05–45.50) versus 45.5 (42.43–47.53), *P* = 0.001) were significantly lower in the severe group than in the mild group. In contrast, the levels of C‐reactive protein (CRP) (*P* = 0.04) and procalcitonin (PCT) (*P* = 0.006), as well as the percentage of neutrophils (*P* = 0.027), were significantly higher in the severe group than those in the mild group, while no significant differences in the levels of white blood cells (WBCs), alanine aminotransferase (ALT), aspartate aminotransferase (AST), and lactate dehydrogenase (LDH) were detected between the two groups (*P* > 0.05). Table 3 provides a comparison of laboratory tests between the two groups.

**TABLE 3:** Comparison of laboratory tests in children with HBoV‐positive mild and severe pneumonia (x̄ ± s or M[Q1, Q3]).

	Severe group (*n* = 47)	Mild group (*n* = 55)	χ^2^/*z*/*t*	*P*
WBCs	11.3 (8.59–14.19)	10.2 (7.34–13.68)	−0.665	0.506
PLT	352.11 ± 154.62	405.91 ± 162.36	–	0.091
Hb	109.02 ± 15.55	115.38 ± 13.21	–	0.028
L	0.41 ± 0.18	0.52 ± 0.20	–	0.003
N	0.52 ± 0.20	0.43 ± 0.20	–	0.027
ALT	26.0 (19.2–38.7)	23.5 (18.0–28.7)	−1.699	0.089
AST	43.6 (34–62)	39.5 (34.53–44.45)	−1.853	0.064
LDH	329.4 (268.5–440.0)	315.2 (280.25–353.55)	−1.230	0.219
ALB	42.5 (38.05–45.50)	45.5 (42.43–47.53)	−3.218	0.001
Elevated PCT	18 (38.3%)	8 (14.5%)	7.528	0.006
Elevated CRP	10 (21.3%)	4 (7.3%)	4.197	0.040

Abbreviations: ALB, albumin; ALT, alanine aminotransferase; AST, aspartate aminotransferase; Hb, hemoglobin; L, lymphocytes; LDH, lactate dehydrogenase; N, neutrophils; PLT, platelet (blood platelets); WBC, white blood cells.

### 3.3 Comparison of pathogen detection between children with severe and mild HBoV‐positive pneumonia

HBoV was mono‐detected in 34 cases (33.3%) and co‐detected in 68 cases (66.7%). There was no significant difference in the severity of the disease between cases where HBoV was the sole pathogen and those where it was co‐detected with other pathogens (*P* > 0.05). Among the 102 HBoV‐positive children, 38 (37.3%) had co‐infections with bacteria, with *Haemophilus influenzae* being the most commonly co‐detected bacterium (17 cases, 16.7%), followed by *Streptococcus pneumoniae* (16 cases, 15.7%) and *Mycoplasma pneumoniae* (10 cases, 9.8%). Viral co‐infections were identified in 47 cases (46.1%), with RSV being the most commonly co‐detected virus (27 cases, 26.5%), followed by PIV (11 cases, 10.8%). No significant differences were noted in pathogen detection between children with severe and mild HBoV‐positive pneumonia. Chest X‐ray and/or CT imaging identified pulmonary consolidation in 13 cases (27.7%) within the severe group, a higher incidence than that in the mild group. Table 4 details the results of auxiliary examinations for both groups.

**TABLE 4:** Comparison of pathogen detection in children with HBoV‐positive mild and severe pneumonia (x̄ ± s or M[Q1, Q3]).

	Severe group (*n* = 47)	Mild group (*n* = 55)	χ^2^/*z*/*t*	*P*
Co‐detection with bacteria	19 (40.4%)	19 (34.5%)	0.375	0.540
*Staphylococcus aureus*	2 (4.3%)	0 (0%)	‐	0.210
*Haemophilus influenzae*	10 (21.3%)	7 (12.7%)	1.334	0.248
*Haemophilus parainfluenzae*	1 (2.1%)	3 (5.5%)	0.123	0.725
*Streptococcus pneumoniae*	6 (12.8%)	10 (18.2%)	0.562	0.453
*Moraxella catarrhalis*	1 (2.1%)	3 (5.5%)	0.123	0.725
*E. coli*	2 (4.3%)	4 (7.3%)	0.417	0.519
Co‐detection with other viruses	22 (46.8%)	25 (45.5%)	0.019	0.891
RSV	12 (25.5%)	15 (27.3%)	0.039	0.843
PIV	4 (8.5%)	7 (12.7%)	0.468	0.494
HAdV	5 (10.6%)	3 (5.5%)	0.361	0.548
IVA	2 (4.3%)	2 (3.6%)	0.000	1.000
HRV	2 (4.3%)	4 (7.3%)	0.417	0.519
*Mycoplasma pneumoniae*	6 (12.8%)	4 (7.3%)	0.355	0.551
Co‐detection	29 (61.7%)	39 (70.9%)	0.967	0.325
Imaging				
Striped or patchy shadow	27 (57.4%)	33 (60.0%)	0.068	0.794
Lung consolidation	13 (27.7%)	4 (7.3%)	7.584	0.006
Pleural effusion	2 (4.3%)	0 (0%)	‐	0.210

Abbreviations: E. coli, Escherichia coli; HAdV, human adenovirus; HRV, human rhinovirus; IVA, influenza virus A; PIV, parainfluenza virus; RSV, respiratory syncytial virus.

### 3.4 Comparison of clinical characteristics of children with mono‐detected and co‐detected HBoV‐positive severe pneumonia

Among the 47 children diagnosed with severe pneumonia, HBoV was the sole detected pathogen in 18 cases (38.3%) but was co‐detected with other pathogens in 29 cases (61.7%). Comparative analysis of the clinical characteristics of the mono‐ and co‐detection groups was conducted. As detailed in Table 5, there were no significant differences in respiratory clinical symptoms or laboratory test results between the two groups.

**TABLE 5:** Comparison of clinical characteristics of children with mono‐detected and co‐detected HBoV‐positive severe pneumonia (x̄ ± s or M[Q1, Q3]).

	Mono‐detection group (*n* = 18)	Co‐detection group (*n* = 29)	χ^2^/*z*/*t*	*P*
Clinical picture				
Cough	18 (100.0%)	29 (100.0%)	–	–
Breathing	11 (61.1%)	16 (55.2%)	0.160	0.689
Shortness of breath	4 (22.2%)	12 (41.4%)	1.815	0.178
Fever	12 (66.7%)	15 (51.7%)	1.014	0.314
Diarrhea	7 (38.9%)	10 (34.5%)	0.220	0.691
Rale	18 (100.0%)	28 (96.6%)	0.634	0.426
Wheezing rale	13 (72.2%)	19 (65.5%)	0.230	0.632
Nasal flaring/Inspiratory retraction	10 (55.6%)	18 (62.1%)	0.196	0.658
Cyanosis	15 (83.3%)	19 (65.5%)	1.762	0.184
Laboratory examination				
WBC	12.00 ± 4.44	11.35 ± 4.23	0.510	0.612
PLT	354.94 ± 125.97	350.34 ± 172.14	0.098	0.922
Hb	114.06 ± 14.62	105.90 ± 15.52	1.790	0.080
L	0.45 ± 0.16	0.40 ± 0.16	1.137	0.262
N	0.49 ± 0.18	0.55 ± 0.18	−1.263	0.213
ALT	25 (19.5–41.0)	29 (21.0–43.1)	−0.164	0.870
AST	40.0 (34.0–46.0)	44.2 (36.8–60.0)	−1.554	0.120
LDH	328.0 (269.2–410.0)	332.0 (278.1–440.6)	−1.641	0.101
ALB	40.77 (37.0–43.5)	42.02 (40.0–45.2)	−1.105	0.269
Imaging				
Catkins or patchy shadows	10 (55.6%)	17 (58.6%)	0.043	0.836
Lung consolidation	5 (27.8%)	8 (27.6%)	–	0.989
Pleural effusion	0 (0%)	2 (6.9%)	–	0.517

Abbreviations: ALB, albumin; ALT, alanine aminotransferase; AST, aspartate aminotransferase; Hb, hemoglobin; L, lymphocytes; LDH, lactate dehydrogenase; N, neutrophils; PLT, platelet (blood platelets); WBC, white blood cells.

### 3.5 Comparison of complications in children with single‐detection and co‐detection HBoV‐positive severe pneumonia

Regarding complications, the mono‐detection group exhibited a higher incidence of atelectasis and bronchiectasis than the co‐detection group, but fewer complications related to the cardiovascular, digestive, and blood systems, as well as electrolyte disorders, although these differences were not significant (Table 6). Notably, the occurrence of neurological complications was significantly higher in the co‐detection group than that in the mono‐detection group (*P* = 0.024). Among these, four cases (8.5%) were detected with HAdV, two cases (4.3%) were detected with RSV, and one case (2.1%) was detected with PIV, suggesting a potential association of HAdV with neurological complications. However, neither mono‐detection nor co‐detection significantly influenced the occurrence of other systemic complications.

**TABLE 6:** Comparison of complications in children with mono‐detected and co‐detected HBoV‐positive severe pneumonia (*n* [%]).

	Mono‐detection group (*n* = 18)	Co‐detection group (*n* = 29)	*P*
Atelectasis or bronchiectasis	4 (22.2%)	3 (10.3%)	0.403
Cardiovascular system	4 (22.2%)	8 (27.6%)	0.744
Digestive system	7 (38.9%)	12 (41.4%)	0.866
Nervous system	0 (0%)	7 (24.1%)	0.024
The blood system	4 (22.2%)	8 (27.6%)	0.744
Electrolyte disturbance	2 (11.1%)	5 (17.2%)	0.692

### 3.6 Risk factor analysis

Clinical factors including length of hospital stay, clinical manifestations, complications, underlying diseases, and laboratory test results from all 102 HBoV‐positive children were subjected to both univariate and multivariate regression analyses. Univariate logistic regression analysis identified several risk factors for HBoV‐positive severe pneumonia, including airway or bronchopulmonary dysplasia, presence of fever, shortness of breath, nasal flaring, inspiratory retractions, wheezing, cardiovascular and hematological complications, and decreased Hb, ALB, lymphocytes (L), and elevated neutrophils (N), CRP, and PCT levels. Multivariate analysis also revealed hypoalbuminemia (odds ratio [OR] = 0.83, 95% confidence interval [CI] = 0.69–0.98) as an independent risk factor for HBoV‐positive severe pneumonia (*P* = 0.030) (as detailed in the Supplementary Data).

## 4 DISCUSSION

Acute lower respiratory tract infection is a major cause of morbidity and mortality in children under 5 years old globally. Respiratory viruses are the primary pathogens causing pediatric respiratory tract infections, with human enterovirus/human rhinovirus (HEV/HRV), HBoV, RSV, and HMPV being the most commonly detected viruses in severe pneumonia cases with a single virus infection.^14^ In a birth cohort study of Australian children aged <2 years, HBoV emerged as the fourth most common virus after HRV, human polyomaviruses WU/KI, and seasonal coronaviruses.^15^ Recent meta‐analysis found that the prevalence of HBoV varies from 2.00% to 45.69%, with a pooled prevalence of 9.57%.^16^ This high prevalence underscores the need for clinicians to give HBoV greater consideration. Although the pathogenicity of HBoV remains a subject of debate due to its frequent co‐detection with other pathogens, fatal cases of severe respiratory tract infection solely attributed to HBoV have been reported. However, studies on the clinical characteristics of cohorts with HBoV‐positive severe pneumonia are limited.

Our study found that 5.88% of severe pneumonia cases were HBoV‐positive. Furthermore, the single detection of HBoV in severe pneumonia cases suggested that HBoV may play a causative role in children with severe acute respiratory infection. This finding differs somewhat from that of Petrarca et al.,^17^ who reported an 11.6% incidence of HBoV positivity in severe cases with respiratory failure. This discrepancy may be due to differences in sample types or detection methods applied. Using next‐generation sequencing (NGS), Moesker et al.^18^ reported a single infection incidence rate of 14% (7/49) in HBoV‐positive patients with severe acute respiratory infection, suggesting that HBoV can cause severe acute respiratory infections in children in the absence of other pathogens. In this study, the independent detection of HBoV in respiratory specimens from children with severe pneumonia further underscores its significant role as a viral pathogen in these cases.

Our results showed that HBoV detection in Chongqing, China, did not exhibit obvious seasonality, with sporadic occurrences throughout the year, although the detection rate in summer was higher, and consistent with previous studies.^19^, ^20^ In contrast, Zhang et al.^6^ reported that the highest prevalence of HBoV occurred in winter in Zhejiang (China), while Silva et al.^21^ reported the highest prevalence of HBoV infection in spring and autumn in Brazil. Furthermore, a previous multicenter prospective study^14^ observed that unlike RSV, which shows similar epidemic patterns in Northern and Southern China, the patterns for HBoV and HAdV differ between these regions. This suggests regional variations in HBoV prevalence, potentially influenced by local climatic factors, underscoring the importance of region‐specific epidemiological data for early identification, prevention, and control. In this study, the typical age range for the HBoV‐infected children was from 6 months to 2 years old, indicating a higher susceptibility in infants with lower immunity, corroborating previous reports.^9^, ^22^ In contrast to RSV, which more commonly infects infants under 6 months, the higher age prevalence for HBoV infection may relate to the presence of maternally derived HBoV antibodies in infants under 6 months. Notably, HBoV seroprevalence in this age group is reported to be as high as 74%.^23^ The varying epidemic seasonality of HBoV in different regions may be related to meteorological conditions and human behavior, as temperature and humidity can influence virus stability in the environment, subsequently impacting viral transmission.

Regarding the clinical characteristics of HBoV‐positive severe pneumonia, our study revealed that cough, fever, shortness of breath, and dyspnea were the most frequently observed symptoms, similar to cases of severe community‐acquired pneumonia (CAP) not associated with HBoV. Consequently, it is challenging to clinically distinguish pneumonia caused by HBoV from that caused by other pathogens based solely on these symptoms. Notably, a higher incidence of diarrhea was observed in children with HBoV‐positive severe pneumonia. Increased wheezing, linked to respiratory hyperresponsiveness and viral infection‐induced inflammatory immune response, was also more prevalent in patients with detected HBoV, as reported in other studies.^9^ The higher incidence of digestive symptoms in HBoV‐positive patients may be related to the HBoV subtype. HBoV is categorized into four types, with HBoV1 primarily causing respiratory diseases and, to a lesser extent, digestive diseases, while HBoV2 and HBoV3 are predominantly associated with gastrointestinal conditions such as acute gastroenteritis.^3^ Due to the extremely low detection rate of HBoV4, reports concerning HBoV4 are relatively scarce in the literature. Our previous research found that HBoV1 is closely related to wheezing episodes in respiratory infections, with wheezing being more common in children with higher viral loads.^19^ Therefore, when children present with common respiratory symptoms like cough and wheezing, especially when accompanied by prominent gastrointestinal symptoms, and taking into account factors like age and seasonality, the possibility of HBoV infection should be considered.

HBoV is often associated with the detection of multiple pathogens. In our study, we observed no significant differences in disease severity between patients with HBoV detection alone and those with concurrent detection of other pathogens. Among those in the severe group, 61.7% had infections involving other pathogens alongside HBoV. The clinical symptoms of severe pneumonia were similar in cases of isolated HBoV infection and in those with co‐detection, suggesting that HBoV alone can also lead to severe pneumonia. Several studies have discussed the co‐infection of HBoV and other viruses and bacteria in children with severe CAP.^19^ Our study detailed the types of pathogens found in conjunction with HBoV, providing more comprehensive reference data for clinical practice. Our results showed that RSV was the most common viral pathogen, followed by HAdV, while *Haemophilus influenzae* was the most common bacterial co‐infectant. Contrary to the findings of Sun et al.,^24^ no significant difference was observed in the severity of HBoV‐positive pneumonia between cases with co‐detection and single detection in the severe and mild groups, supporting the pathogenic role of HBoV. In severe pneumonia, the incidence of neurological complications was higher in the co‐detection group than that in the single detection group, indicating a greater likelihood of neurological symptoms under co‐infection, especially with HAdV. No significant differences were found in WBC, ALT, AST, and LDH levels between the HBoV‐positive severe and mild groups. However, a marked decrease in Hb and ALB levels was observed in HBoV‐positive severe patients. Notably, hypoalbuminemia emerged as an independent risk factor for disease severity, which may be linked to the increase in vascular endothelial permeability, ALB exudation, and Hb consumption caused by infection.^25^, ^26^ Previous analyses of viral pneumonia patients admitted to the pediatric ICU identified hypoalbuminemia as a significant predictor of mortality.^27^ Severely ill children may experience complications such as anemia, cardiac insufficiency, toxic encephalopathy, and atelectasis. Similar to complications observed in severe acute respiratory syndrome coronavirus 2 (SARS‐CoV‐2) infections,^24^, ^25^ these issues may be related to dysregulation in the response of the body to severe viral infection, leading to systemic organ dysfunction. This dysfunction may be related to an imbalance in host immune homeostasis, uncontrolled cytokine storm, and associated organ damage.^28^, ^29^ This suggests that HBoV‐positive severe pneumonia may progress rapidly, underscoring the importance of early identification and intervention for effective prevention and control.

Previous studies have identified various risk factors for HBoV‐positive severe pneumonia, including prematurity, history of recurrent infection, history of assisted ventilation, immunodeficiency, congenital heart disease, and interstitial lung disease.^3^ In this study, the presence of airway or bronchopulmonary dysplasia was identified as a potential risk factor for severe infection, consistent with previous research.^30^ Additionally, failure to receive the BCG vaccine before the onset of illness was also identified as a risk factor for the exacerbation of HBoV pneumonia. Studies have shown that BCG vaccination can promote the innate immune response of the host, inhibit viral replication, reduce viral load, and reduce inflammation and symptoms.^31^ BCG vaccination not only helps prevent tuberculosis but also reportedly reduces respiratory infections, including those caused by RSV, influenza virus A (IVA), and herpes simplex virus 2 (HSV2), by 70%.^31^ In addition, vaccinations for pneumonia‐related diseases (e.g., diphtheria, tetanus, pertussis [DTP], measles, and BCG) can effectively reduce pneumonia mortality.^32^ No significant differences were found in congenital heart disease and malnutrition in this study, which may be related to the small sample size, suggesting the need for further studies with a larger sample size. All nine cases of severe respiratory tract infection involving HBoV were found to have viremia, indicating a strong link between HBoV viremia and acute primary infection as well as moderate to severe disease. The presence of HBoV viremia, which can disseminate to other body parts, may also be connected to the development of gastrointestinal symptoms. Christensen et al.^33^ found viremia in 18 out of 40 children exhibiting respiratory symptoms, with 88.8% of these cases being children younger than 2 years, while viremia was absent in the control group. Additionally, viremia was more prevalent in patients with a high viral load in NPAs, indicating that viremia may be a contributing factor to severe respiratory infections, consistent with our previous study.^34^ Integrating the analysis of underlying diseases and risk factors in children with severe pneumonia from this study, preterm birth, wheezing, history of previous infection, underlying pulmonary diseases such as airway or bronchopulmonary dysplasia, and viremia may be risk factors for severe pneumonia in cases positive for HBoV.

This study has several limitations to be mentioned. First, this study was retrospective in nature and was constrained in small sample size, partly due to the lower prevalence of HBoV than RSV in severe acute respiratory infections. Our clinical samples were also limited to nasopharyngeal secretions and did not include the collection of blood samples, sequencing of HBoV isolates, or examination of HBoV1 mRNA transcriptional activity,^35^ which are essential for distinguishing between new infection events and the reactivation of latent virus following primary infection. The detection of HBoV viremia and serological analysis would have provided stronger support for the diagnosis of acute HBoV infection.^7^ Second, it has been reported that HBoV can persist in the tonsil lymphoid germinal centers of asymptomatic children.^36^ This prolonged shedding complicates the diagnosis of acute infection, and the persistence, latency, and reactivation mechanisms of HBoV are still not fully understood. While the clinical sensitivity of qualitative PCR detection for HBoV DNA is higher than that of HBoV mRNA detection, its specificity is lower.^37^, ^38^ Consequently, the association between viremia and severe pneumonia caused by HBoV‐positive cases is not yet clearly established. Future studies should investigate this relationship using blood samples and a combined nasopharyngeal secretion mRNA detection approach.

## 5 CONCLUSIONS

This study describes the epidemiological and clinical characteristics of severe pneumonia in HBoV‐positive children, underscoring the importance of HBoV in pediatric severe pneumonia. Notably, our findings support the hypothesis that HBoV is a causative pathogen of severe pneumonia rather than a simple passenger. HBoV‐positive severe pneumonia cases were primarily found in children aged between 6 months and 2 years. While HBoV‐positive severe pneumonia occurred sporadically throughout the year, the detection rate was somewhat higher in summer. Instances of HBoV detection without accompanying pathogens in children were associated with certain complications, such as atelectasis or bronchiectasis. Various factors, such as airway or bronchopulmonary dysplasia, absence of BCG vaccination, viremia, and hypoproteinemia, were identified as potential risk factors for HBoV‐positive severe pneumonia. The single identification of HBoV in respiratory specimens from children with severe pneumonia suggests its role as a key viral pathogen in such hospitalized cases. Overall, this study strengthens evidence of the pathogenicity of HBoV in severe acute respiratory infection in children. Therefore, HBoV testing should be considered for routine diagnosis of children with severe respiratory tract infections.

## AUTHOR CONTRIBUTIONS

LJ conceptualized and designed the study, and drafted the initial manuscript. YZY, HY, and WJH collected the data and carried out the initial analyses. RL provided important comments while developing the manuscript, reviewed and revised the manuscript. LEM and ZN took overall responsibility for the design, implementation and analysis of the study. All authors approved the final manuscript as submitted and agree to be accountable for all aspects of the work.

## CONFLICT OF INTEREST STATEMENT

The authors declare no conflicts of interest.

## ETHICS STATEMENT

This study was approved by the Medical Ethics Committee of the Children's Hospital Affiliated to Chongqing Medical University (Ethics Approval [Research] No [217‐1], 2019).

## Supporting information

Table S8

## Data Availability

The data that support the findings of this study are available from the corresponding author upon reasonable request.
