# Supplementary material for: Clinical characteristics and risk factors of severe bocavirus‐positive pneumonia in children and a literature review
Source: Pediatr Discov. 2025 Feb 6;3(4):e2523. doi: 10.1002/pdi3.2523 (PMC12753018; doi:10.1002/pdi3.2523)
Supplement: Supplementary file 1 — Table S8 [file PDI3-3-e2523-s001.docx]

Table8 Analysis of risk factors for HBoV-positive severe pneumonia

|  | Univariate analysis | | | Multivariate analysis | | |
| --- | --- | --- | --- | --- | --- | --- |
|  | OR | 95%CI | *P* | OR | 95%CI | *P* |
| Tachypnea | 0.19 | 1.72-15.5 | 0.003 |  |  |  |
| Fever | 2.56 | 1.15-5.70 | 0.022 |  |  |  |
| Nasal flaring/Inspiratory retraction | 9.58 | 10.12-25.64 | 0.000 |  |  |  |
| Wheezing rale | 2.38 | 1.06-5.35 | 0.036 |  |  |  |
| Cardiovascular complications | 3.43 | 1.11-10.61 | 0.032 |  |  |  |
| Hematologic complications | 3.43 | 1.11-10.61 | 0.032 |  |  |  |
| Airway or bronchopulmonary dysplasia | 4.69 | 1.21-18.20 | 0.026 |  |  |  |
| Decreased Hb | 0.97 | 0.94-0.99 | 0.031 |  |  |  |
| Decreased L | 0.04 | 0.01-0.37 | 0.004 |  |  |  |
| Elevated N | 9.16 | 1.24-67.61 | 0.030 |  |  |  |
| Elevated CRP | 3.45 | 1.01-11.84 | 0.049 |  |  |  |
| Elevated PCT | 3.65 | 1.41-9.46 | 0.008 |  |  |  |
| Decreased ALB | 0.85 | 0.77-0.95 | 0.003 | 0.83 | 0.69-0.98 | 0.030 |
